# Identification of *Hyalomma* Ticks on Migratory Birds in Poland During the 2023 and 2024 Spring Seasons

**DOI:** 10.3390/life15081311

**Published:** 2025-08-19

**Authors:** Klaudia Bylińska, Jan Rapczyński, Paweł Górski, Oliwia Obuch-Woszczatyńska, Damian Pietrzak, Karol Korzekwa, Małgorzata Krzyżowska, Piotr Bąska

**Affiliations:** 1 Laboratory of Parasitology, Military Institute of Hygiene and Epidemiology, 01-163 Warsaw, Poland; klaudia.bylinska@wihe.pl (K.B.); oliwia.obuch@wihe.pl (O.O.-W.); damianpietrzak23@gmail.com (D.P.); 2 Division of Pharmacology and Toxicology, Department of Preclinical Sciences, Institute of Veterinary Medicine, Warsaw University of Life Sciences, 02-786 Warsaw, Poland; 3 Foundation for Nature Conservation “On Wings”, 02-640 Warsaw, Poland; janrapczynski@gmail.com; 4 Forestry Student Scientific Association, Ornithological Section, Warsaw University of Life Sciences, 02-787 Warsaw, Poland; 5 Division of Parasitology and Parasitic Diseases, Department of Preclinical Sciences, Institute of Veterinary Medicine, Warsaw University of Life Sciences, 02-786 Warsaw, Poland; pawel_gorski@sggw.edu.pl; 6 Department of Medical and Environmental Microbiology, Military Institute of Hygiene and Epidemiology, 01-163 Warsaw, Poland; karol.korzekwa@wihe.pl (K.K.); malgorzata.krzyzowska@wihe.pl (M.K.)

**Keywords:** *Hyalomma rufipes*, *Hyalomma marginatum*, ticks, CCHFV, *Rickettsia*, *Rickettsia aeschlimannii*, Poland, Europe

## Abstract

Ticks from the *Hyalomma* genus have recently garnered public attention in countries in Northern and Central Europe, as they are transported by migratory birds and might have established stable populations due to climate warming. The main threat associated with *Hyalomma* ticks is their ability to transmit Crimean–Congo hemorrhagic fever virus (CCHFV), which can be fatal in up to 40% of cases. Here, we collected *Hyalomma* ticks from migratory birds during annual ringing actions in the spring seasons of 2023 and 2024. Four ticks were found on birds from the *Acrocephalus* genus and two on *Hippolais icterina*. The ticks were examined for the presence of *Rickettsia* spp., *Babesia microti*, *Babesia divergens*, *Coxiella burnetii*, *Borreliella burgdorferi* (s. l.), *Anaplasma phagocytophilum*, West Nile virus, and CCHF virus (CCHFV). The collected *H. rufipes* specimens were negative for tested pathogens, except for two ticks collected in 2024, which were positive for *Rickettsia aeschlimannii*. The data show that *Hyalomma* ticks are efficiently transported on birds preferring reedbeds or deciduous trees. The possibility of the occurrence of CCHF or rickettsiosis (induced by *R. aeschlimannii*) is currently assessed as low. Nevertheless, we have shown the transfer of *Hyalomma* ticks to Poland and indicated the need for careful future epidemiological monitoring of the presence of *Hyalomma* ticks.

## 1. Introduction

Ticks are small ectoparasites that feed on blood, and around 900 species inhabiting various climate zones have been described [1].

They are the subject of interest for both human and veterinary medicine since they are vectors of multiple diseases. Depending on species and geographical region, they can spread the causative agents of viral tick-borne encephalitis (TBE), African swine fever (ASF) [2], Crimean–Congo hemorrhagic fever (CCHF), Kyasanur Forest Disease (KFD), anaplasmosis, Lyme disease, rickettsioses [3], ehrlichiosis [4], tularemia [5], Q fever [6], babesiosis [7], theileriosis, cytauxzoonosis, hepatozoonosis, and equine piroplasmosis [8]. The most predominant and endemic species in Northern and Central Europe is *Ixodes ricinus*, but *I. arboricola*, *I. ariadnae*, *I. frontalis*, *I. lividus*, *I. persulcatus*, *Dermacentor reticulatus*, *Rhipicephalus sanguineus*, *Haemaphysalis concinna*, and *H. punctata* may also occur [9]. The southern regions of Europe, such as Spain, the Balkans, Italy, and Turkey, are highly populated with *Hyalomma* spp. (*H. marginatum* and *H. rufipes).* These ticks infest birds as larvae, undergo engorgement and molting into nymphs, which can be transported over long distances and in this manner may be introduced to the northern regions of Europe [10]. Upon transportation by migratory birds during the spring season, the larva molts into an adult and feeds on larger mammals, i.a., humans. Until recently, *Hyalomma* ticks were considered to survive only for a few months in Northern Europe, being incapable of adapting to harsher winters. However, adult *H. marginatum* and *H. rufipes* specimen have recently been found in England [11], Germany [12], Netherlands [13], and Sweden [14], allowing us to hypothesize that *Hyalomma* genus may have adapted to the new environment and established stable populations in Central and Northern Europe [12], posing a threat of CCHF [15]. This raises concerns about importing not only CCHFV, which poses a significant threat to humans, but also pathogens dangerous to animals. *Hyalomma* ticks are vectors for *Theileria equi*, *Babesia caballi*, and *T. annulata*, which are causative agents of equine piroplasmosis [8] and tropical theileriosis in cattle [8], respectively. These conditions have not been reported as problems in Central and Northern Europe [16,17,18]. Although the risk of new diseases being introduced via *Hyalomma* ticks transported by migratory birds is considered low [13], the increased number of *Hyalomma* ticks encountered raises the need for continuous epidemiological surveillance, especially in view of global warming, facilitating the development of stable populations. This is a new challenge for medical and veterinary services. To fill the knowledge gap regarding the epidemiological situation in Poland, we investigated migratory birds in two spring seasons (2023 and 2024) to determine the presence of *Hyalomma* spp. and the potential pathogens they may carry.

## 2. Materials and Methods

### 2.1. Collection of Ticks

All ticks were collected from migratory birds during routine ringing action performed in Góra Kalwaria (51.97° N, 21.26° E) during the 2023 spring season and in Góra Kalwaria, Nowa Dęba (50.5° N, 21.8° E), and Wicie (54.5° N, 16.46° E) during the 2024 spring season. Tick specimens collected from birds were preliminarily identified based on their morphology [19] as *Hyalomma* species and placed in separate tubes, and then transported to the laboratory. Upon arrival, the ticks were frozen at −80 °C until use.

### 2.2. Nucleic Acid Isolation

Total Nucleic Acid (tNA) was isolated from ticks using either the Tick RNA/DNA Purification Kit (Eurx, Gdansk, Poland) or the DNA/RNA Extraction kit (Eurx, Gdansk, Poland). All isolated nucleic acids were quantified and characterized using NanoDrop (Thermo Fisher Scientific, Waltham, MA, USA). cDNAs from tick RNA collected in 2023 and 2024 were synthesized using GoScript™ Reverse Transcriptase (Promega Corporation, Madison, WI, USA).

### 2.3. Molecular Identification of Species and Sequence Analysis

**Figure 1:**
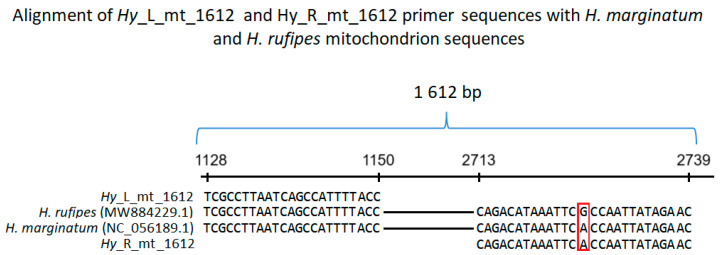
Alignment of *Hy*_L_mt_1612 and *Hy*_R_mt_1612 primer sequences with fragments of *H. marginatum* (NC_056189.1) and *H. rufipes* (MW884229.1) mitochondrial sequences. The reverse primer (*Hy*_R_mt_1612) sequence is shown as reverse complementary. The numbers on the horizontal line represent nucleotides in *H. marginatum* (NC_056189.1) and *H. rufipes* (MW884229.1) mitochondrial sequences. The red frame highlights a mismatch between the analyzed sequences.

Primers based on *H. marginatum* (NC_056189.1) and *H. rufipes* (MW884229.1) mitochondrion sequences were designed as follows: Hy_L_mt_1612 (TCGCCTTAATCAGCCATTTTACC) and Hy_R_mt_1612 (GTTCTATAATTGGTGAATTTATGTCTG). Primer Hy_L_mt shows 100% homology to both *H. marginatum* and *H. rufipes* mitochondrion sequences (Figure 1). Primer Hy_R_mt shows 100% homology to *H. marginatum* and one mismatch to *H. rufipes* mitochondrion sequence. Hy_L_mt and Hy_R_mt amplify a 1612 bp long fragment of both *H. marginatum* and *H. rufipes* mitochondrial sequences (Figure 1). PCR amplification of DNA isolated from each tick was performed in a total volume of 20 µL. The reaction mixture consisted of 1× PCR Master Mix (K0172, Thermo Fisher Scientific Baltics UAB, Vilnius, Lithuania), 0.3 µM Hy_L_mt_1612, 0.3 µM Hy_R_mt_1612, and 1 µL of DNA solution. The cycling conditions were as follows: 95 °C (6 min), 40 × (95 °C—30 s, 55 °C—1 min 30 s, 72 °C—2 min 30 s), 72 °C (25 min) (2720 Thermal Cycler, Life Technologies Holdings Pte Ltd., Singapore). The reactions were resolved on 1% agarose gel stained with Simply Safe (E4600-01, Eurx Sp. z o.o., Gdansk, Poland).

The sequences of the amplified mtDNA fragments from each isolate were deposited in GenBank. The entries in GenBank showing the highest similarity to each of the sequences were identified using BLASTn (Word size: 28, Match/Mismatch score: “1, −2” and linear Gap Costs, database: core_nt, taxid: *Hyalomma* (taxid no. 34625)), followed by retrieval from GenBank and alignment construction using MultiAlin [20]. The simple versions of the alignments (showing only differing residues) were visualized manually and are presented in the main text. The full version was visualized using Jalview 2.11.4.1 [21] and is presented in the Supplementary Material due to its size.

For phylogenetic analyses, a consensus sequence was constructed using MultiAlin. The consensus was used as input for BLASTn analyses (using the parameters mentioned above). The sequences showing 100% coverage were retrieved and aligned with the sequences from *Hyalomma* isolates using Clustal Omega (default parameters) [22]. Based on the alignment, a phylogenetic tree was constructed using MEGA software (12.0.11) [23], with the Neighbor-joining method with 1000 bootstrap replicates.

### 2.4. Detection of Pathogens in Ticks

qPCR TaqMan assays containing primers and probes for detecting *Rickettsia* spp. (Ba07922462), *Babesia microti* (Pr07922374), *Babesia divergens* (Pr07922376), *Coxiella burnetii* (Ba06439618), *Borreliella burgdorferi* (Ba07922354), *Anaplasma phagocytophilum* (Ba07922353), and West Nile virus (Vi04329496) were purchased from Thermo Fisher Scientific (Waltham, MA, USA). The assays were mixed with GoTaq^®^ Probe qPCR Master Mix (Promega Corporation, Madison, WI, USA) according to the manufacturer’s instructions. PCR amplification of the DNA/RNA was performed to detect the abovementioned pathogens. RNA was transcribed into cDNA using MLV reverse transcriptase (Thermo Fisher Scientific, Waltham, MA, USA). The reactions were performed in a total volume of 20 µL using QuantStudio™ 5 Real-Time Thermocycler (Life Technologies Holdings Pte Ltd, Singapore) with the following cycling conditions: 95 °C (2 min), 40 × (95 °C—3 s, 60 °C—30 s).

#### 2.4.1. Rickettsia Species

Samples positive for rickettsial DNA were subjected to further determination of the species. Fragments of 23S–5S interspacer region (ITS) from the ticks were amplified using 23S_forward (GATAGGTCGGGTGTGGAAGCAC) and 23S_reverse (GGGATGGGATCGTGTGTTTCAC) primers, whereas fragments of the outer membrane protein OmpAIV gene were amplified using RR_190-5125 (GCGGTTACTTTAGCCAAAGG) and cRR_190-6013 (TCTTCTGCGTTGCATTACCG) primers [24]. The reaction mixtures consisted of 1× PCR Master Mix (K0172, Thermo Fisher Scientific Baltics UAB, Vilnius, Lithuania), a mix of forward and reverse primers (0.3 µM each), and 19–35 ng of genomic DNA isolated from the ticks. The cycling conditions were as follows: 95 °C (6 min), 40× (95 °C—20 s, 57 °C—30 s, 72 °C—1 min), 72 °C—15 min for amplification of ITS fragment, and 95 °C (6 min), 40 × (95 °C—20 s, 50 °C—30 s, 72 °C—1 min 30 s), 72 °C—15 for OmpA fragment. All reactions were performed in a total volume of 20 µL. The reactions were resolved in 2% (ITS) or 1% (OmpA) agarose gels stained with Simply Safe (E4600-01, Eurx Sp. z o.o., Gdansk, Poland), followed by extraction of the appropriate bands: ~380 bp for ITS and ~880 bp for OmpA. The DNA fragments were extracted from the gel using Agarose-Out DNA Purification Kit (E3540, Eurx Sp. z o.o., Gdansk, Poland) and subjected to Sanger sequencing.

#### 2.4.2. Detection of CCHFV

Two sets of primers, based on Orthonairovirus haemorrhagiae (CCHFV) isolate WJQ16206 segment L sequence (MG659722.1), were designed to detect CCFFV. Set no. 1 consisted of primers CCHFV_1267_L (GATGAGCAAAAGGAGGACTGAG) and CCHFV_1267_R (CATTAAGGACAGAGCATGATTTAGTG). The primes spanned MG659722.1 sequence regions 10,287–10,299 and 11,544–11,519, and amplified a 1267 bp long product. Set no. 2 consisted of primers CCHFV_1685_L (AGCTAGGGCACAAGTTGCTAAC) and CCHFV_1685_R (ATCTTTCCATTTTTTATGGTCTGTCC). The primes spanned MG659722.1 sequence regions 941–962 and 2625–2600, and amplified a 1685 bp long product. PCR reactions were performed on cDNA matrices using PCR Master Mix (2×) (K0172, Thermo Fisher Scientific Baltics UAB, Vilnius, Lithuania) with the following reaction mixture composition: PCR Master Mix (1×), 0.3 µM CCHFV_1267_L (or CCHFV_1685_L), 0.3 µM CCHFV_1267_R (or CCHFV_1685_R), and 1 µL of cDNA, in a total volume of 20 µL. The cycling conditions were as follows: 95 °C (6 min), 40 × (95 °C—30 s, 55 °C—1 min 30 s, 72 °C—2 min 30 s), 72 °C—15 min. The reactions were resolved in 1% agarose gel stained with Simply Safe (E4600-01, Eurx Sp. z o.o., Gdansk, Poland).

We also used primers and probes previously designed by Sas et al. [25], as shown in Table 1, with modification of the quenchers. In the original paper, the BHQ 1 quencher was used, whereas we used TAMRA. To validate the assay in our laboratory and check its efficiency, a synthetic oligonucleotide (standard) was designed based on the CCHFV genotype IV sequence (Table 1). To test the compatibility of the primer and probe sequences and the designed standard with our equipment and reagents, the reaction was performed as follows: the total mixture volume was 12 µL and consisted of 1× GoTaq^®^ Probe qPCR Master Mix (Promega Corporation, Madison, WI, USA, Cat. No. A6102), CXR (30 nM) (Promega Corporation, Madison, WI, USA) as a passive reference, primers CCHF-I-f, CCHF-I-r, CCHF-II-f, CCHF-II-r, CCHF-III-f, CCHF-III-r, CCHF-IV-f, CCHF-IV-r, CCHF-V-f, CCHF-V-r, CCHF-VI-f, CCHF-VI-r (100 nM each), primers CCHF-deg-f and CCHF-deg-r (400 nM each), and probes CCHF-probe-1 and CCHF-probe-2 (100 nM each). The triplicate reactions were performed on a 10-fold serial dilution (10^6^—0 copies per reaction) of the standard in QuantStudio™ 5 Real-Time Thermocycler ((Life Technologies Holdings Pte Ltd, Singapore)) using the following program: 95 °C—2 min, 45 × (95 °C—15 s, 55 °C—30 s, 60 °C—1 min). The results were satisfactory; thus, the assay was also used to detect CCHFV genetic material in synthesized cDNA (1 µL) from the ticks.

## 3. Results

### 3.1. Collection of Hyalomma Ticks

During the 2023 and 2024 spring seasons, 1156 and 1554 birds were examined in Góra Kalwaria, resulting in the identification of 342 and 118 ticks, respectively. Góra Kalwaria is located on the left bank of the middle course of the Vistula River and is covered by fruit-bearing shrubs and willow thickets (Table 2). In 2024, 106 and 167 birds were inspected for ticks in Nowa Dęba and Wicie, resulting in the identification of 10 and 53 ticks, respectively (Table 2). Wicie is characterized by the presence of a reed bed and low fruit-bearing shrubs at the base of the spit that separates the freshwater lake from the Baltic Sea coast. Nowa Dęba contains extensive reed beds and shrubs in a large complex of fish farming ponds.

Altogether, six *Hyalomma* ticks were collected from the birds and identified (Table 2). On average, we identified three tick specimens on migratory birds in each year. Four ticks were collected in Góra Kalwaria from *Acrocephalus palustris* and *Hippolais icterina*, one in Wicie (*Acrocephalus arundinaceus*), and one in Nowa Dęba (*Acrocephalus schoenobaenous*). Two ticks, collected on 24 May, were found on the same bird (*Hippolais icterina*). The isolates’ names are assigned based on the first letter of the town and the order in which they were found (Table 2).

### 3.2. Computational Analyses

**Figure 2:**
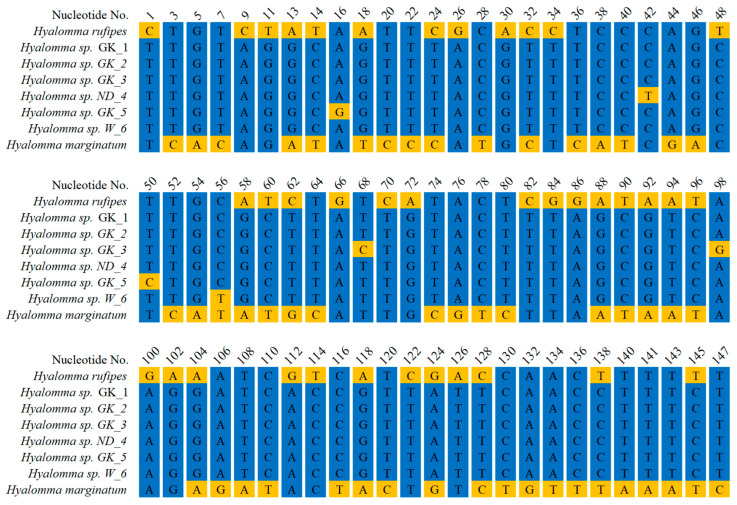
Alignment of mtDNA fragments from *H. marginatum* and *H. rufipes* with the corresponding fragments from *Hyalomma* ticks collected in Poland. Isolates: GK_1) Góra Kalwaria, 24 May 2023 (PQ563190); GK_2) Góra Kalwaria, 24 May 2023 (PQ563191); GK_3) Góra Kalwaria (PQ563192), 25 May 2023; ND_4) Nowa Dęba, 14 May 2024 (PQ563193); GK_5) Góra Kalwaria, 14 May 2024 (PQ563194); W_6) Wicie, 24 May 2023 (PQ563195). The sequences of DNA fragments from *H. rufipes* (KY457528.1) and *H. marginatum* (NC_056189.1) were retrieved from GenBank. The total length of aligned sequences is 1562 bp, with 1519 identical residues across all sequences. The presented alignment depicts only the residues that differ across the sequences.

mtDNA fragments of all specimens were amplified, sequenced, and deposited in GenBank under the following Accession Numbers: *Hyalomma* sp. GK_1 (PQ563190), GK_2 (PQ563191), GK_3 (PQ563192), ND_4 (PQ563193), GK_5 (PQ563194), and W_6 (PQ563195). BLAST analyses revealed that all six sequences show high similarity to *H. rufipes* (KY457528.1), with the following percentages of identical nucleotides: 97.57%, 97.57%, 97.44%, 97.5%, 97. 44%, and 97.5% for GK_1, GK_2, GK_3, ND_4, GK_5, and W_6 isolates, respectively (Supplementary Figure S1). Multiple alignment of the sequences with corresponding fragments from *H. rufipes* and *H. marginatum* revealed 95.2% (1487/1, 562) of identical residues among all the sequences and 4.8% (75/1562) of residues with various nucleotides (Figure 2, Supplementary Figure S1). GK_1 and GK_2 ticks were collected from the same bird (*Hippolais icterina*), and their sequences were the only ones to show 100% identity across this comparison (Figure 2, Supplementary Figure S1). The constructed alignment (Figure 2) depicts only the varying nucleotides, while the full alignment is presented in Supplementary Figure S1.

**Figure 3:**
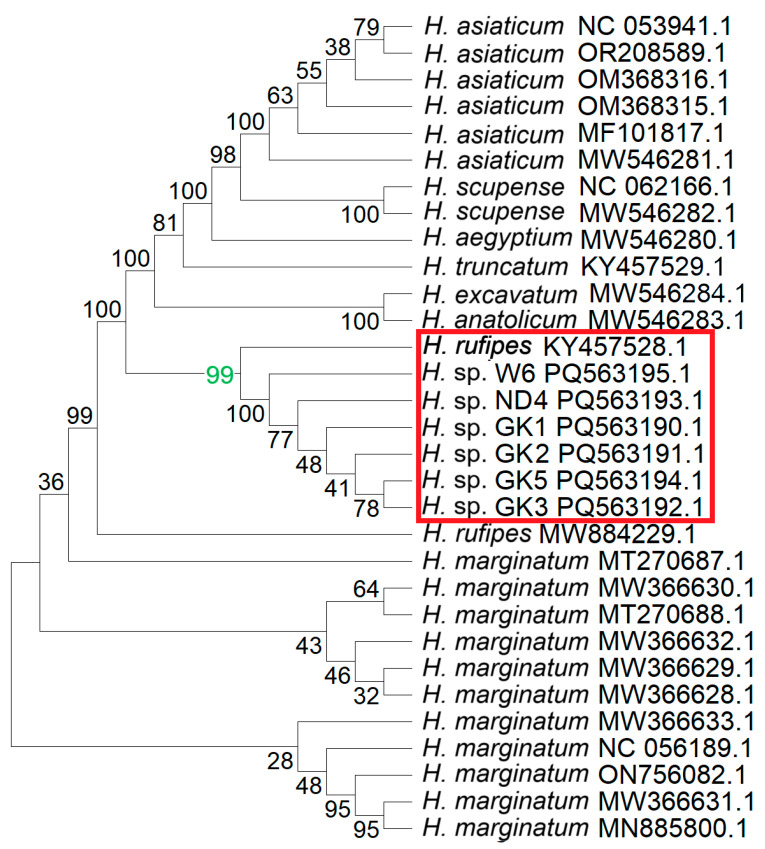
Results of phylogenetic analysis of fragments of the sequenced mtDNA and other *Hyalomma* species. The consensus of the sequenced mtDNA fragments was constructed, followed by BLAST analyses, which allowed for the retrieval of sequences from GenBank showing 100% coverage to the sequences from *Hyalomma* isolates GK_1, GK_2, GK_3, ND_4, GK_5, and W6. The sequences were analyzed using the Neighbor-Joining method, with 1000 bootstrap replicates. The resulting Bootstrap Consensus Tree is shown. Numbers at the nodes represent bootstrap support values, indicating the percentage of replicates in which a given clade was recovered. The value 99 (green) strongly indicates that GK1, GK2, GK3, ND4, GK5, W6, and *H. rufipes* all belong to the same clade (red frame).

The BLAST analyses of the consensus sequences resulted in the identification of 25 sequences in GenBank belonging to the *Hyalomma* genus, showing 100% coverage with the consensus (Figure 3). The phylogenetic analysis revealed the highest homology of the investigated sequences to the *H. rufipes.*

### 3.3. Detection of Pathogens in Ticks

**Figure 4:**
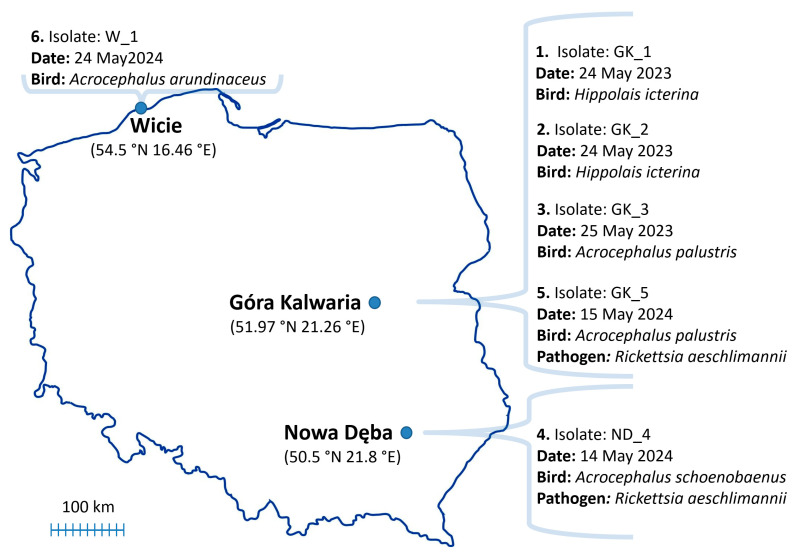
Geographic distribution of collected *Hyalomma rufipes* ticks that tested positive for pathogens. Ticks were ordered according to the date of collection. All the ticks were negative for CCHFV, West Nile virus, *Anaplasma* spp., *Babesia* spp., *Coxiella* spp., and *Borrelia* spp. Two ticks (ND_4 and GK_5) were positive for *R. aeschlimannii*.

All the analyzed ticks were negative for *B. microti*, *B. divergens*, *C. burnetii*, *B. burgdorferi* (s. l.), *A. phagocytophilum*, CCHFV, and West Nile virus. One tick collected in Nowa Dęba (ND_4) and one in Góra Kalwaria (GK_5) were positive for *Rickettsia* spp. (Figure 4).

#### 3.3.1. Identification of Rickettsia Species

**Figure 5:**
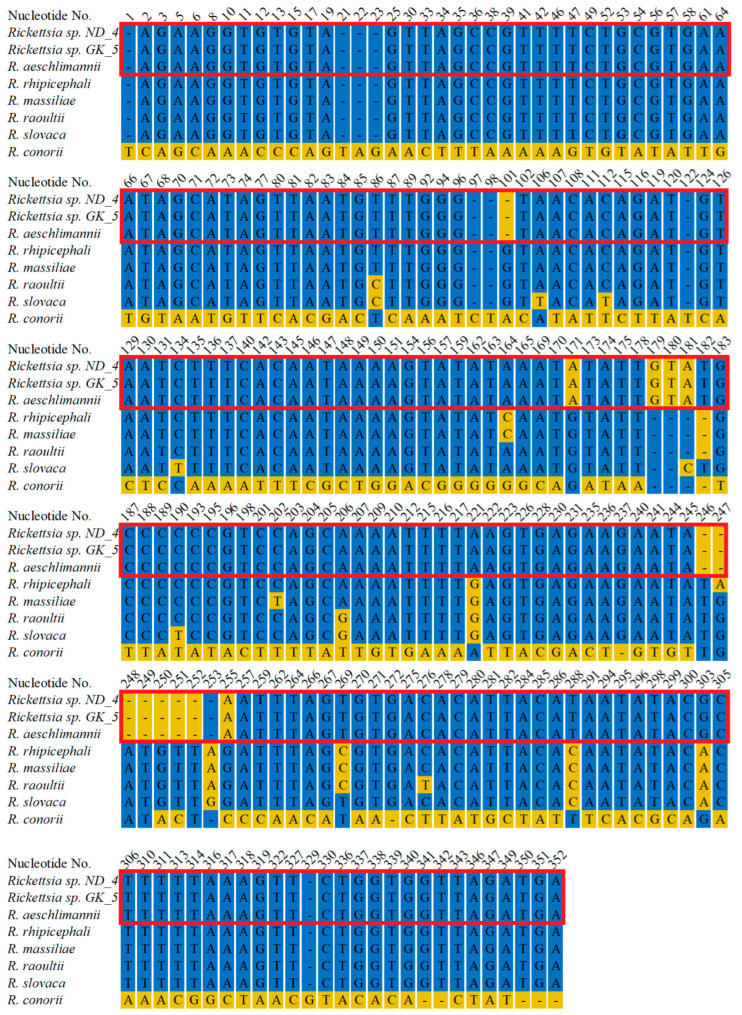
Alignment of rDNA fragments from *Rickettsia* detected in ticks collected in Nowa Dęba (ND) and Góra Kalwaria (GK) with corresponding fragments from other *Rickettsia* species. *Rickettsia* sp. ND_4, the sequence of a DNA fragment from *Rickettsia* sp. collected from a tick in Nowa Dęba (14 May 2024) (GenBank No. PV335514). *Rickettsia* sp. GK_5, the sequence of a DNA fragment from *Rickettsia* sp. collected from a tick in Góra Kalwaria (15 May 2024) (GenBank No PV335515). The sequenced fragments were searched against GenBank using the BLAST algorithm. Representative sequences from closely related species were retrieved, followed by construction of an alignment using MultiAlin and manual visualization. *R. aeschlimannii* (GenBank No. MW295947.1), *R. rhipicephali* (GenBank No. CP003342.1), *R. massiliae* (GenBank No. PP263042.1), *R. raoultii* (GenBank No. MG974041.1), *R. conorii* (GenBank No. CP098324.1). The red frame indicates 100% identity between the ND_4 and GK_5 sequences with *R. aeschlimannii.* The presented alignment depicts only the residues that differ across the sequences.

**Figure 6:**
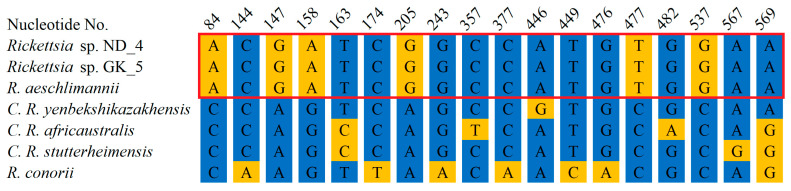
Alignment of genomic DNA encoding outer membrane protein A (OmpA) fragments from *Rickettsia* detected in ticks collected in Nowa Dęba (ND) and Góra Kalwaria (GK) with corresponding fragments from other *Rickettsia* species. *Rickettsia* sp. ND_4—the sequence of a DNA fragment from *Rickettsia* sp. detected in a tick from Nowa Dęba (14 May 2024) (GenBank No. PV335516). *Rickettsia* sp. GK_5—the sequence of a DNA fragment from *Rickettsia* sp. detected in a tick from Góra Kalwaria (15 May 2024) (GenBank No. PV335517). The sequenced fragments were searched against GenBank using the BLAST algorithm. Representative sequences from closely related species were retrieved, followed by construction of an alignment using MultiAlin and manual visualization. *R. aeschlimannii* (GenBank No. OR687042.1), *C. R. yenbekshikazakhensis* (GenBank No. MG974005.1), *C. R. africaustralis* (GenBank No. KT835203.1), *C. R. stutterheimensis* (GenBank No. KT835243.1), *R. conorii* (GenBank No. OR148302.1). The red frame indicates 100% identity between the ND_4 and GK_5 sequences with *R. aeschlimannii.* The presented alignment depicts only the residues that differ across the sequences.

Further, we amplified rRNA and OmpA encoding fragments to determine the pathogen species. We amplified 335 bp long rDNA fragments from both ND_4 and GK_5 isolates, sequenced, and deposited them in GenBank under the following numbers: PV335514 and PV335515. The sequencing revealed their 100% identity to each other and the corresponding fragment of *R. aeschlimannii* (MW295947.1) and a lower identity to other *Rickettsia* species: 94.8%, 94.19%, 93.9%, 93.6% and 93.9% to *R. rhipicephali* (CP003342.1), *R. massiliae* (PP263042.1), *R. raoultii* (MG974041.1), *R. slovaca* (OZ002747.1), and *R. conorii* (CP098324.1), respectively (Figure 5, Supplementary Figure S2). Amplification of DNA encoding OmpA fragments resulted in 849 bp products, which were also sequenced using the Sanger technique. In the case of the ND_4 isolate, the full-length fragment was sequenced and deposited in GenBank (PV335516). Despite numerous attempts, we were unable to obtain credible results for the entire 849 bp fragment from the GK_5 isolate. To obtain high-quality sequences of the 5′ and 3′ ends of the amplified fragments, we designed two new primers (Rick_OMP_5_end: ATTACCACCTGACTTAGCCGC and Rick_OMP_3_end: AATGACACCGCTACAGGAAGC); however, the results were still unsatisfactory. In this case, a shorter fragment (704 bp) was deposited in GenBank (PV335517). BLAST analysis of the ND_4 isolate sequence revealed its 100% identity to *R. aeschlimannii* (OR687042.1) corresponding fragment and 99.92%, 98.82%, 98.82%, and 98.35% identical residues with *Candidatus Rickettsia yenbekshikazakhensis* (MG974005.1), *C. R. africaustralis* (KT835203.1), *C. R. stutterheimensis* (KT835243.1), and *R. conorii* (OR148302.1), respectively (Figure 6, Supplementary Figure S3). Analyses of the sequenced fragment from GK_5 isolate also showed 100% identical residues with *R. aeschlimannii*, as well as 99.01%, 98.58%, 98.72%, and 98.15% identity with C. *R. yenbekshikazakhensis*, *C. R. africaustralis*, *C. R. stutterheimensis*, *and R. conorii*, respectively (Figure 6, Supplementary Figure S3). Based on these results, both *Rickettsia* isolates were classified as *R. aeschlimannii* species.

#### 3.3.2. CCHFV Detection

**Figure 7:**
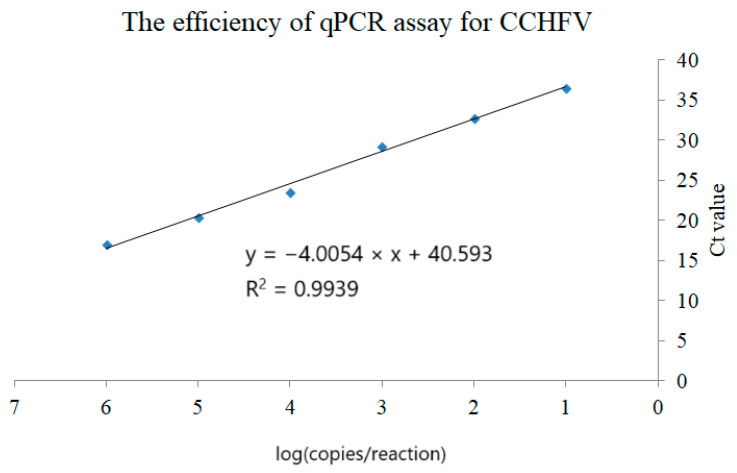
Efficiency of qPCR amplification of CCHFV showing the dependency between the number of copies and the Ct value. The “x” axis represents logarithmic values from copy number per reaction, and the “y” axis represents corresponding medians from the three replicates. The calculated trend line R^2^ coefficient shows linear dependency. The assay could detect one copy of the standard (10^0^) per reaction, but only in one of three replicates (not included in the figure).

Two RT-PCR assays based on self-designed primers and one RT-qPCR [25] detecting six genotypes of CCHFV were used to examine ticks for the presence of the virus. Both RT-PCR assays found no bands corresponding to the desired molecular mass. After initial experiments confirmed that the primers and probes designed by Sas et al. [25] and the synthetic standard were compatible with our equipment and reagents (Figure 7), we performed analyses to detect CCHFV nucleic acid in the investigated samples. The reaction was positive for three replicates of the standard concentration, ranging from 10^6^ to 10 copies per reaction. We also detected one copy per reaction, but only in one of three replicates. Linear dependency showed a high R^2^ coefficient (0.9939) (Figure 6). All the tested samples (GK_1, GK_2, GK_3, ND_4, GK_5, and W_6) were negative.

## 4. Discussion

We confirmed the presence of *Hylomma* ticks on migratory birds in Poland in the spring seasons of 2023 and 2024. There are two approaches to identifying species: based on morphology or using DNA analyses. Both methods are useful but have disadvantages. Morphological identification of engorged nymphs is challenging [26]. For accurate identification of *Hyalomma* ticks, it is suggested that nymphs be allowed to molt into adults [27]. However, the transstadial transmission of pathogens remains uncertain [28], potentially leading to negative results in adult specimens even when the corresponding nymphs are infected. The molecular method is more precise, but in the case of *Hyalomma* differentiation, it may be impeded by high intra-genus DNA homology and cryptic hybridization within the genus [29]. We combined these methods as described by Uiterwijk et al. [13]. Following preliminary morphological identification, we analyzed fragments of mtDNA spanning 1527 (99.2%) nucleotides of the cox-1 gene, which is widely used in species determination, and assigned the specimens to the *H. rufipes* genus belonging to the *H. marginatum* complex, which contains *H. marginatum*, *H. turanicum*, and *H. rufipes* [26], which are considered a new emerging threat to public health in Central and Northern Europe. Their low occurrence has been previously noted in Poland. Retrospective analyses of the Natural History Department (Museum of Upper Silesia in Bytom, Poland) collection revealed the occurrence of one *H. marginatum* specimen in 1935 and three in 1943 [30]. The subsequent two cases were described in 1979 [31] and 2010 [30] and were associated with migratory birds. Another species, *H. aegyptium*, was noted on imported turtles in the 1980s (or earlier) [32], and 78 specimens were identified on reptiles imported to Poland in 2003–2007 [33]. However, the increased number of recently collected specimens in Europe and the new threat of establishing stable populations (associated with climate change) raise concerns about introducing new pathogens, especially CCHFV [34], to European regions. To address the epidemiological situation of potential CCHFV introduction, we examined migratory birds in Poland for the presence of *Hyalomma* ticks and their ability to transfer CCHFV. *Hyalomma* ticks were identified on four migratory bird species—*A. palustris*, *A. arundinaceus*, *A. schoenobaenus*, and *H. icterina*—in the spring of 2023 and 2024. The results correspond to data from other European countries, revealing the presence of *Hyalomma* ticks on *Acrocephalus* spp. in Germany [35], the Czech Republic, Slovakia [10], and the Netherlands [13]. This indicates the existence of a correlation between a niche occupied by a bird and the probability of importing *Hyalomma* ticks. *A. arundinaceus* and *A.schoenobaenus* share similar habitat preferences; they occur primarily in reedbeds, where *A. palustris* frequently occurs as well, but can also be found in low bushes and meadows. *H. icterina* prefers deciduous trees and bushes. Each species migrates from Poland predominantly between August and September towards warmer African regions. The wintering grounds of *A. schoenobaenus* and *A. arundinaceus* are spread across sub-Saharan Africa, apart from the Somali Peninsula and the far southern part of the continent (Republic of South Africa, Botswana). *H. icterina* has a smaller wintering range that extends from the Democratic Republic of Congo to the eastern parts of South Africa. The wintering range of *A. palustris* is limited to southeastern Africa, specifically Mozambique, Zimbabwe, and the Eastern parts of South Africa. All four species avoid the Southernmost parts of Africa [36]. They spend the winter months in areas endemic for *Hyalomma* ticks [37] and CCHFV [38], raising the possibility of the disease being imported to the European territory. *A. schoenobaenus*, *A. arundinaceus*, and *H. icterina* may migrate to Europe across the Sahara Desert and the Mediterranean Sea, potentially passing through the Balkans, Hungary, and Slovakia on their way to Poland. *A. palustris* is more likely to travel along the west coast of Africa, through Somalia, Ethiopia, Sudan, and Egypt; however, the precise routes for individual specimens remain unknown. Laboratory experiments have indicated that immature *H. rufipes* may remain attached to the host for up to 28 days [39]. Thus, the collected ticks could have attached to the birds in any of the countries mentioned above and may originate from either Africa or Southern Europe.

The number of *Hyalomma* ticks was 6 per 2983 birds (0.2%), which can be considered low. Similar research was performed by Capek et al. (2014), who identified 30 *Hyalomma* specimens per 1172 birds in the Czech Republic and Slovakia [10]; 68.75% of birds infested with *Hyalomma* ticks were found at a latitude of 47.52° N in Slovakia, with a decreasing prevalence of *Hyalomma* ticks on birds in progressively higher latitudes [40]. Moreover, although researchers found 15 ticks in the Czech territory, 8 and 4 of the specimens were found on two birds [10], revealing an uneven distribution of ticks and indicating problems with analyses of such results. Dividing the tick number by the number of investigated birds may not be an appropriate method for analyzing the data. An additional factor to consider is the presence of the Tatra Mountains, which form a natural geographic barrier between Slovakia and Poland. This topographic obstacle may compel migratory birds to prolong their stopover duration prior to crossing, potentially increasing the likelihood of tick detachment and thereby reducing the number of tick specimens transported into Poland. This hypothesis is potentially supported by a similar survey in the Benelux, where one *Hyalomma* specimen was found per 375 singing birds, which is comparable with our results (0.27%) [41]. Unfortunately, this comparison cannot be confirmed since the authors provided information on birds trapped from 2012 to 2014 without providing monthly trapping results. Therefore, it is not possible to determine whether the tick was associated with bird migration. [41]. Similarly, other reports indicating the presence *of Hyalomma* in Central Europe (Germany and the Netherlands) cannot be quantitatively compared to our results since they were not epidemiological surveys targeting birds. The *Hyalomma* ticks delivered to the researchers were obtained from single specimens [13,35]. Considering all the data, it is difficult to predict the significance of the transportation of six ticks that were identified during the survey. The exact number is low, and considering all the migrating birds may lead to a change in the results; nevertheless, despite all the doubts, discrepancies, and difficulties in statistical interpretation, these results clearly show that the transportation of *Hyalomma* ticks occurs. Moreover, it is almost certain that this phenomenon has occurred during seasonal bird migration for hundreds or thousands of years, resulting in no adverse effects. However, the mild winters and global warming provide grounds for gaining a deeper insight into this phenomenon. Another question that needs to be answered is the geographical origin of the identified *Hyalomma* specimens. Although it is difficult to determine, collecting such data would be beneficial for assessing the risk of potential CCHF cases, since the clinical course of the infection may depend on the strain and origin of the virus [42]. During this research, we found no *Hyalomma* specimens carrying CCHFV, corresponding to the results of field surveys in Germany [12] and the Netherlands [13]. However, the annual transfer of CCHFV-positive *Hyalomma* ticks to Northern and Central Europe is almost certain, since only a negligible number of specimens from the billions of migrating birds [43] can be examined. Even if CCHFV-positive *Hyalomma* are introduced, the risk of spreading the disease is low, since, as discussed above, few ticks are currently introduced to the environment, and the very dispersed population would substantially decrease the possibility of finding a tick mating partner, efficiently preventing the spread of the virus [13]. Nevertheless, monitoring is necessary since, at local levels, *Dermacentor* spp. and *Rhipicephalus* spp. can transfer the CCHFV [44]. Cuadrado-Matías et al. [45] recently identified CCHFV in questing *I. ricinus* in Spain, suggesting potential adaptation, at least at the local level, of CCHFV transmitted by endemic European ticks. Both the development of stable populations and the potential adaptation need further investigation, even though the risk of CCHF occurrence is low; on the other hand, low probability does not mean “negligible probability”. The probability of the coronavirus (causative agent of COVID-19) crossing the intraspecies barrier was also very low, yet it happened. Species migration and adaptation to northern regions may accelerate with global warming; it is difficult to predict whether CO_2_ emissions will be reduced and whether global warming will be reversed. These processes do not occur ad hoc, and our data do not indicate a high number of transported *Hyalomma specimens;* however, the six ticks identified here should be considered proof of their transportation into Polish territory. The results may be a valuable component in the mathematical modelling of the spread of *Haylomma* (and its pathogens) during the age of accelerated data analysis revolution (big data and AI). We do not postulate a rapid CCHF outbreak but encourage the collection and analysis of data that are beneficial for public health. It would be beneficial to inform physicians or Doctors of Veterinary Medicine (DVMs) about a slightly increased risk of CCHF or animal piroplasmosis, respectively, in cases of tick bites.

We also examined ticks for other pathogens and obtained negative results for *B. microti*, *B. divergens*, *C. burnetii*, *B. burgdorferi* (s.l.), and *A. phagocytophilum*. However, two ticks collected in 2024 were positive for *Rickettsia* spp., a finding similar to *Hyalomma* ticks identified in Austria [46] and Germany [12,35]. The Rickettsiae are Gram-negative bacteria spread by ticks and induce febrile tick-borne illnesses known as spotted fevers [47], which may result in meningitis, perimyocarditis, facial palsy, and sudden deafness [37]. The mortality rate can be as high as 30% without prompt antibiotic treatment [48]. Although doxycycline efficiently prevents the development of severe complications [49], the introduction of new strains and genetic material recombination with the native species poses a threat of developing unpredictable effects. The species we identified, *R. aeschlimannii*, was described for the first time in 1997 [50]. It can be spread by *H. marginatum*, *H. rufipes* [51], *H. aegyptium* [52], *H. punctata*, *R. bursa*, *R. sanguineus*, *R. turanicus*, and *I. ricinus* [53]. The first case of *D. reticulatus* bearing *R. aeschlimannii* was recently published [54]. Infected people suffer from spotted fever symptoms, including fever, a maculopapular rash, eschars, hyponatremia, hypokalemia, and lesions [55,56]. The bacteria can also infect animals, as recently proven by Erol et al. [57], who confirmed their presence in cat blood samples in Turkey. Epidemiological surveys confirmed the presence of *R. aeschlimannii* in Germany [35], the Netherlands [13], and Poland [54]. In the first two cases, it was associated with *Hyalomma* ticks, whereas in Poland, the bacteria were identified in *D. reticulatus*, which cannot be imported by birds. This raises a question regarding the prevalence of *R. aeschlimannii* in Northern and Central European ticks and its potential to be spread by endemic species, mainly *I. ricinus*. Although *I. ricinus* was found to bear this bacterium, the rate of infected ticks is lowest (0.23%) compared to other species, with *H. marginatum* having the highest rate (5.86%). On the other hand, *I. ricinus* tick numbers in Northern and Central Europe dramatically exceed those of *Hyalomma* spp., leaving an open question regarding *R. aeschlimannii*’s potential to induce spotted fever in Europe. However, due to the lack of confirmed cases, the risk should currently be assessed as low.

## 5. Conclusions

*Hyalomma* ticks are a new threat to Central and Northern Europe. They are imported by migratory birds and may have adapted to Central and Northern European climates. Their presence is associated with the threat of introducing new pathogens and novel strains of the existing ones. We collected six *H. rufipes* ticks from migratory birds, mainly from the *Acrocephalus* genus, over two spring seasons (2023 and 2024). None of the ticks were vectors for CCHFV, but two were positive for *R. aeschlimannii*. Although the risk of an increase in CCHF and *R. aeschlimannii* infections is considered low, the data show the importance of continuous monitoring of imported ticks for public health and the prevention of new disease outbreaks in Central and Northern Europe.

## Figures and Tables

**Table 1:** The sequences of primers, probes, and the synthetic oligonucleotide used for CCHF RNA detection. The used primers amplified six CCHFV genotypes. Forward and reverse primers for each genotype are assigned “f” or “r”, respectively, in the primer name. The number in the name of each primer stands for the genotype it amplifies. Primers CCHF-deg-f and CCHF-deg-r can amplify all genotypes. A synthetic oligonucleotide used as a standard was designed based on the genotype IV sequence; it contains sequences that bind to primers CCHF-IV-f (orange) and CCHF-IV-r (blue), as well as the probe CCHF-probe-2 (green) in reverse complement orientation. The primers and probes (but not the standard) sequences were obtained from previously published data [25].

Primer	Sequence
CCHF-I-f	CAAGAGGCACTAAAAAAATGAAGAAGGC
CCHF-I-r	GCAACAGGGATGGTTCCAAAGCAAAC
CCHF-II-f	CAAGGGGYACCAARAAAATGAAGAAGGC
CCHF-II-r	GCYACRGGGATGGTTCCRAAGCAGAC
CCHF-III-f	CAAGAGGTACCAAGAAAATGAAGAAGGC
CCHF-III-r	GCCACGGGGATTGTCCCAAAGCAGAC
CCHF-IV-f	CAAGGGGTACCAAGAAAATGAAGAARGC
CCHF-IV-r	GCCACAGGGATTGTTCCAAAGCAGAC
CCHF-V-f	CAAGGGGGACCAARAAAATGAAAAAGGC
CCHF-V-r	GCAACAGGGATTGTTCCAAAGCAGAC
CCHF-VI-f	CAAGGGGCACCAAGAAAATGAAGAAAGC
CCHF-VI-r	GCTACAGGAATTGTCCCAAAGCAGAC
CCHF-deg-f	CAAGGGGKACCAAGAAAATGAARAAGGC
CCHF-deg-r	GCMACAGGGATTGTYCCAAAGCAGAC
**Probe**	**Sequence**
CCHF-probe-1	6-FAM-ATCTACATGCACCCTGCYGTGYTGACA-TAMRA
CCHF-probe-2	6-FAM-TTCTTCCCCCACTTCATTGGRGTGCTCA-TAMRA
**Standard**	CAAGGGGTACCAAGAAAATGAAGAAGGCACTCTTGAGCACCCCAATGAAGTGGGGGAAGAAGCTTTTGGGTGTCTGCTTTGGAACAATCCCTGTGGC

**Table 2:** Results of the epidemiological survey showing the presence of *Hyalomma* on birds in Poland. The research was conducted in three locations (Góra Kalwaria, Nowa Dęba, and Wicie) in two Spring seasons (2023 and 2024). The results show the number of examined birds, the number of ticks found, the number of identified *Hyalomma* ticks, and the bird species on which the *Hyalomma* specimens were found. The isolates’ names are assigned based on the first letter of the town and the order in which they were found. *—two *Hyalomma* ticks were found on one bird, *H. icterina* specimen, n.a.—analysis not performed.

Examined Site		2023			2024		
Name and Coordinates		Number of Birds	Number of Ticks	*Hyalomma* Tick; Bird Species; Date; Isolate	Number of Birds	Number of Ticks	*Hyalomma* Tick; Bird Species; Date; Isolate
Góra Kalwawia	51.96° N 21.26° E	1156	342	3 H. icterina * 24 May 2023 GK_1, GK_2 *A. palustris* 25 May 2023 GK_3	1554	118	1 *A. palustris* 25 May 2025 GK_5
Nowa Dęba	50.5° N 21.28° E	n.a.	n.a.	n.a.	106	10	1 *A. schoenobaenous* 14 May 2024 ND_4
Wicie	54.5° N 16.46° E	n.a.	n.a.	n.a.	167	53	1 *A. arudinaceus* *24 May 2024* W_6

## Data Availability

The original contributions presented in this study are included in the article. Further inquiries can be directed to the corresponding author.

## Supplementary Materials

The following supporting information can be downloaded at: https://www.mdpi.com/article/10.3390/life15081311/s1.
