# Supplementary material for: Identification of Hyalomma Ticks on Migratory Birds in Poland During the 2023 and 2024 Spring Seasons
Source: Life (Basel). 2025 Aug 19;15(8):1311. doi: 10.3390/life15081311 (PMC12387227; doi:10.3390/life15081311)
Supplement: Supplementary file 1 [file life-15-01311-s001.zip › Supplementary figure captions.pdf]

Supplementary Figure S1. Alignment of mtDNA fragments from H. marginatum and H. rufipes with the corresponding fragments from Hyalomma ticks collected in Poland. Isolates: GK_1) Góra Kalwaria, 24 May 2023 (PQ563190); GK_2) Góra Kalwaria, 24 May 2023 (PQ563191); GK_3) Góra Kalwaria (PQ563192), 25 May 2023; ND_4) Nowa Dęba, 14 May 2024 (PQ563193); GK_5) Góra Kalwaria, 14 May 2024 (PQ563194); W_6) Wicie, 24 May 2023 (PQ563195). The sequences of DNA fragments from H. rufipes (KY457528.1) and H. marginatum (NC_056189.1 were retrieved from GenBank. The total length of aligned sequences is 1,562 bp long with 1,519 identical residues for all of the sequences. The green tables show the identity of corresponding sequences from H. rufipes and H. marginatum to the sequenced fragments from the investigated isolates (GK_1, GK_2, GK_3, ND_4, GK_5, and W_6).

Supplementary Figure S2. Alignment of rDNA fragments from Rickettsia detected in ticks collected in Nowa Dęba (ND) and Góra Kalwaria (GK) with corresponding fragments from other Rickettsia species. Rickettsia sp. ND_4- the sequence of a DNA fragment from Rickettsia sp. collected from a tick in Nowa Dęba (15 May 2024) (GenBank No. PV335514). Rickettsia sp. GK_5- the sequence of a DNA fragment from Rickettsia sp. collected from a tick in Góra Kalwaria (15 May 2024) (GenBank No PV335515). The sequenced fragments were searched against GenBank using BLAST algorithm. The representative sequences from closely related species were retrieved, followed by the alignment construction using MultiAlin and visualization with Jalview . R aeschlimannii (GenBank No. MW295947.1), R rhipicephali (GenBank No. CP003342.1), R. massiliae (GenBank No. PP263042.1), R raoultii (GenBank No. MG974041.1), R. conorii (GenBank No. CP098324.1). The green tables show the identity of particular sequences from homology species to Rickettsia sp. (ND_4) and Rickettsia sp. (GK_5). The red frame indicates 100% identity of the ND_4 and GK_5 sequences to R. aeschlimannii.

Supplementary Figure S3. Alignment of genomic DNA encoding outer membrane protein A (ompA) fragments from Rickettsia detected in ticks collected in Nowa Dęba (ND) and Góra Kalwaria (GK) with corresponding fragments from other Rickettsia species. Rickettsia sp. ND_4 - the sequence of a DNA fragment from Rickettsia sp. detected in a tick from Nowa Dęba (15 May 2024) (GenBank No. PV335516). Rickettsia sp. GK_5- the sequence of DNA fragment from Rickettsia sp. detected in a tick from Góra Kalwaria (15 May 2024) (GenBank No. PV335517). The sequenced fragments were searched against GenBank using BLAST algorithm. The representative sequences from closely related species were retrieved, followed by the alignment construction using MultiAlin and visualization with Jalview. R. aeschlimannii (GenBank No. OR687042.1), C. R. yenbekshikazakhensis (GenBank No. MG974005.1), C. R. africaustralis (GenBank No. KT835203.1), C. R. stutterheimensis (GenBank No. KT835243.1), R. conorii (GenBank No. OR148302.1). The green tables show the identity of particular sequences from homology species to Rickettsia sp. (ND_4) and Rickettsia sp. (GK_5). The red frame indicates 100% identity of the ND_4 and GK_5 sequences to R. aeschlimannii.
