# Supplementary figures and images for: Identification of Hyalomma Ticks on Migratory Birds in Poland During the 2023 and 2024 Spring Seasons
Source: Life (Basel). 2025 Aug 19;15(8):1311. doi: 10.3390/life15081311 (PMC12387227; doi:10.3390/life15081311)

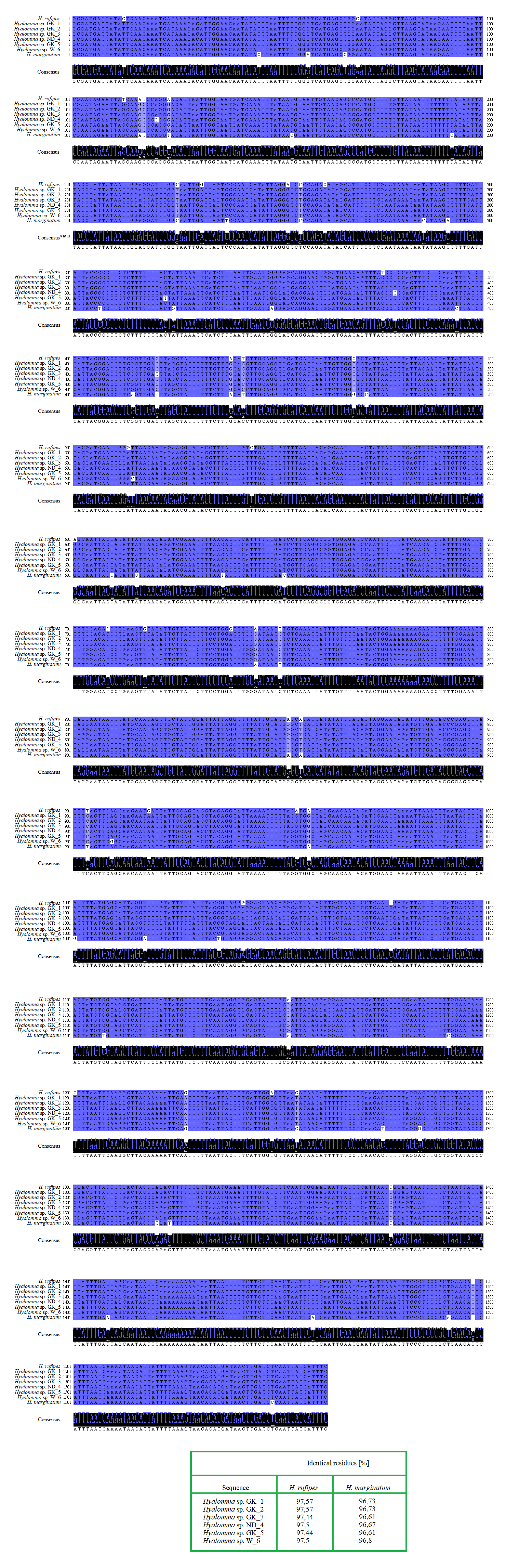

Supplement: Supplementary file 1 [file life-15-01311-s001.zip › Supplementary figure 1.tif]

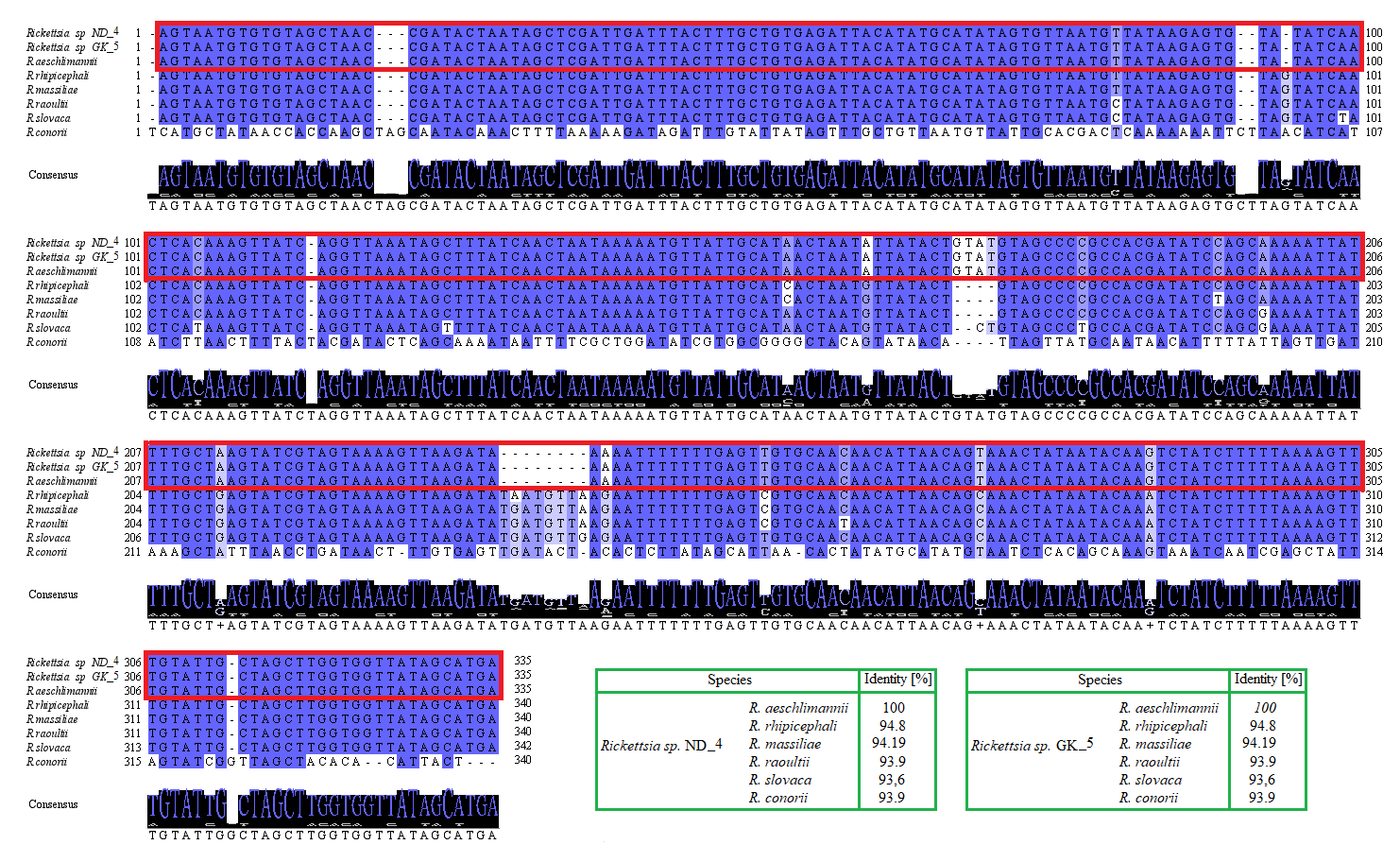

Supplement: Supplementary file 1 [file life-15-01311-s001.zip › Supplementary figure 2.tif]

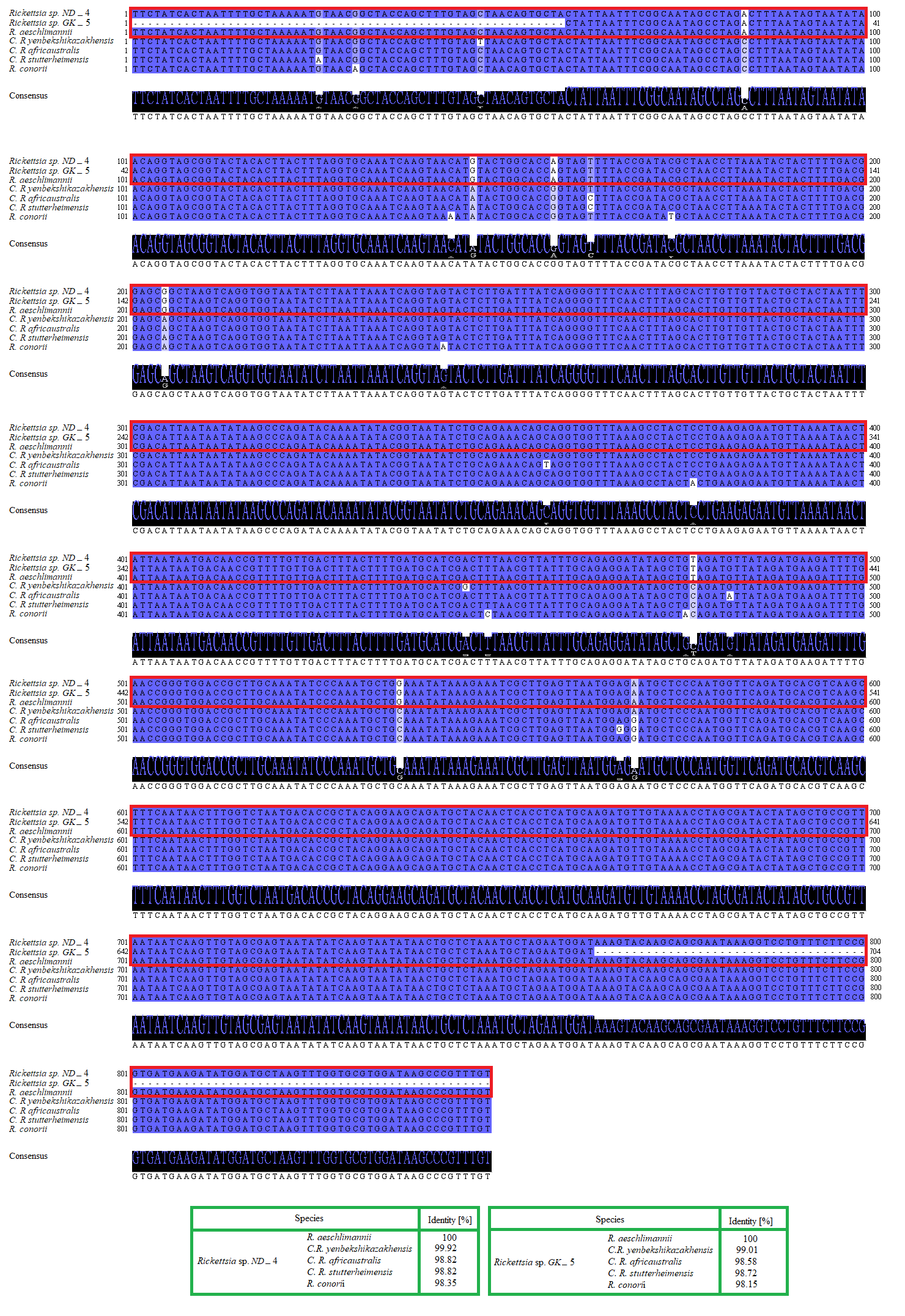

Supplement: Supplementary file 1 [file life-15-01311-s001.zip › Supplementary figure 3.tif]
